# Adaptive modelling approach for predicting causes of death: insights from verbal autopsy data in Tanzania

**DOI:** 10.1093/inthealth/ihaf123

**Published:** 2025-11-17

**Authors:** Mahadia Tunga, James Chambua, Juma Lungo

**Affiliations:** Department of Computer Science and Engineering, College of Information and Communication Technologies, University of Dar es Salaam, P.O. Box 33335 Dar es Salaam, Tanzania; Department of Computer Science and Engineering, College of Information and Communication Technologies, University of Dar es Salaam, P.O. Box 33335 Dar es Salaam, Tanzania; Department of Computer Science and Engineering, College of Information and Communication Technologies, University of Dar es Salaam, P.O. Box 33335 Dar es Salaam, Tanzania

**Keywords:** Bayesian model, cause of death prediction, VA machine learning model, VA model, verbal autopsy, WHO VA questionnaire

## Abstract

**Background:**

The World Health Organization (WHO) has approved the use of a verbal autopsy (VA), a survey-based approach to generate out-of-hospital causes of death (CoDs). Through this study, an adaptive Bayesian networks machine learning model was developed and tested. The model is scalable and adaptable for predicting new causes as the dataset expands.

**Methods:**

The 2016 WHO questionnaire was used to collect data from Iringa, Tanzania, and data augmentation was performed using the Synthetic Minority Oversampling Technique for nominal features to increase the dataset size and reduce bias in the CoD classification. The model development was guided by a CoD decision flow that integrates essential factors and steps for accurate CoD prediction. To our knowledge, no previous study has provided this operational guide for VA cause of death prediction.

**Results:**

The model was evaluated using accuracy, sensitivity, specificity and F1 score metrics and compared with Support Vector Machine and Naïve Bayesian models. Results indicated an average accuracy of 97%, specificity of 97%, sensitivity of 94% and F1 score of 94%, which are superior compared with Naïve Bayesian and Support Vector Machine models.

**Conclusions:**

The reported performance of the developed model demonstrates the potential for this model to enhance VA-based CoD data by integrating a machine learning approach with physician expertise. The results highlight the effectiveness of combining Bayesian networks with physician Symptom Cause Information as a valuable tool in advancing the performance of CoD predictions.

## Introduction

According to the World Health Organisation (WHO),^1^ globally only 50% of deaths are certified with known causes of death (CoDs). However, 50% is insufficient to reflect society’s morbidity conditions. To improve the CoD statistics, the WHO has devised a survey-based verbal autopsy (VA) method.^2^ As a WHO tool, the VA is already being administered in >45 low- and middle-income countries (LMICs). Data generated through VAs are already regarded as useful in determining CoD and helps in making data-driven, informed policy and healthcare interventions.^3^ In practice, the VA is administered to relatives or those aware of the recent death to describe the deceased’s medical history.^4^

Since the VA is being administered in different countries, a gold standard is required to guide its use and ensure accurate determination of CoD.^5^ A gold standard is a database comprising multifarious deaths whose causes have been certified by medical professionals.^6^ Even though this gold standard database does exist, it is inaccessible to LMICs, where most deaths occur without medical care. In most cases, only one-third of deaths in LMICs have a medically certified CoD, and only half of those have a CoD record that can be used for health policy development and planning.^3,6^ These challenges have motivated CoD prediction automation through computer-coded verbal autopsy (CCVA).^2,8–11^

The CCVA approaches use statistical and machine learning (ML) algorithms to determine CoD and thus do not require extensive use of gold standards or medical professionals.^12^ The ML algorithms can explicitly learn and improve their performance as the dataset expands. Today, the availability of ML models is increasing, and they are becoming more effective as proxies for emulating physician coding. The major challenge, however, has been their limited performance.^1^ Therefore, this study proposed an approach that addresses this limitation by developing a model that can predict CoD by combining the strengths of ML algorithms and physician knowledge.

### VA CoD computation algorithms

Several studies employed ML algorithms to compute CoD. The three widely utilized and publicly available algorithms are InterVA5, InSilicoVA and Tariff.^1^ These three algorithms are mostly used for assigning individual CoDs, as presented in the next sections.

The InterVA model uses a naïve Bayesian algorithm to estimate the CoD based on three conditional probabilities: a symptom given in a CoD, a symptom assigned by medical experts and probabilities extracted directly from the CoD.^1^ Performance-wise, the InterVA was reported as having a sensitivity of 0.43 and an accuracy of 0.71 on data from a Million Death Study.^13^

The InSilicoVA employs a Bayesian hierarchical algorithm fitted using a Gibbs sampling algorithm.^14^ The algorithm incorporates information regarding the presence and absence of VA indicators and the conditional probability of each VA indicator for deaths attributed to a specific CoD. The reported mean sensitivity was 0.341 across 34 CoD categories for individual records and 0.85 for cause-specific mortality fraction accuracy.^1^

The Tariff model utilizes a sum of weighted scores called tariffs to determine the most probable CoD. The score for each possible CoD is the weighted sum of different tariffs, each calculated from the value of a specific indicator, which in most of the cases is a symptom or risk factor. The CoD is determined by a strong signal for each cause.^6^ The Tariff model was reported to have an average accuracy of 0.770 for adults, while sensitivity varied for a specific CoD.^6^

While the three algorithms discussed above are the most widely used ones, there are other algorithms that are worthy of mention. The King–Lu model provides a general CoD distribution instead of an individual CoD. Using conditional probability distributions, the King–Lu model computes the general CoD distribution for 13 categories.^15^ The reported accuracy was 0.96 on the Indian Million Death Study dataset,^14^ however, its sensitivity varied for various causes of death.^6^ Also, the King–Lu model does not compute individual CoDs and it does not perform well with small sample sizes.^1^

Jeblee is another model that used the Indian Million Death Study dataset to develop a model that used the narrative portion of the WHO’s VA questionnaire data to compute CoD and achieved an accuracy of 0.908 and sensitivity of 0.662 for 48 CoD categories.^1^ The limitation of the model is that it includes background information irrelevant to the CoD.

This study embarked on developing an adaptive ML prediction model for individual CoD assignments using the WHO’s approved VA questionnaire data with a self-check mechanism that verifies the class size before making CoD predictions. This makes it possible to predict unseen courses as the dataset expands. The model was constructed based on a Bayesian network algorithm and physician knowledge. The choice of a Bayesian algorithm was twofold. First, it uniquely combines expert knowledge with data to deduce complex phenomena.^16–19^ Second, its discrete nature transparently analyses multiclass phenomena such as disease diagnosis.^19–21^

## Methods

### Verbal autopsy questionnaire

The VA data used in this study was collected using the 2016 WHO VA questionnaire and it was administered under the supervision of the Ministry of Health (MoH) of Tanzania. The VA questionnaire comprises a set of structured and unstructured questions. The structured questions are mainly statistical features, which are either categorical or quantitative. The unstructured questions are narratives that describe the patients’ history as explained by the descendants’ caretakers.^14^ The 2016 WHO VA tool was developed to harmonize various existing VA standards and has the advantage of being flexible to add more variables of interest wherever necessary. In Tanzania, the 2016 WHO VA questionnaire was not used as it is, instead a minor customization was made to cater to local needs. Specifically, the WHO questionnaire originally contained 481 questions, however, the MoH introduced 9 extra questions related to health insurance coverage and the location of the interviewer. To match Tanzania’s local context and demands, the questionnaire was adapted and translated into Swahili.^22^ The data were captured using an Open Data Kit (ODK) Collect app that feeds in data directly to the MoH’s servers. ODK Collect is a free, open-source Android app used for collecting and compiling data, particularly in fields like public health, global development and environmental research. The data collection app allows users to create custom digital forms, gather various data types (text, numeric, multimedia) and submit data securely to a server or cloud storage.

### Data analysis

The study used secondary data from the MoH for the Iringa region in Tanzania. Iringa is one of Tanzania’s 31 administrative regions, with a population of 1 149 481. The VA data used in this study were collected from five sites: Iringa Municipal, Kilolo District, Mufindi District, Mafinga Town and Iringa District (Table 1). The death distribution in Mafinga District and Iringa Municipal Council, being <10%, could introduce bias into the model. The similarities of geographic conditions and symptoms/signs of disease patterns in Iringa reduced the risk of model underfitting. The final dataset included 2509 deaths covering all five councils of the region. The training and test data were split in an 80%:20% ratio. In total, 2007 deaths were used to train the model and 502 deaths were used to evaluate model performance.

**Table 1.** Comparison of death distribution by districts.

District	Adults, n (%)
Iringa District Council	843 (34)
Iringa Municipal Council	146 (6)
Kilolo District Council	956 (38)
Mafinga Town Council	111 (4)
Mufindi District Council	453 (18)
Total	2509

Using associations and the identify_nominal_columns functions from the Python dython.nominal libraries, 185 CoDs were identified and referred to as class labels. Based on the heuristic ML approach for multiclass outputs, it is recommended that each class should have at least 1% examples of the class’s total sample size.^23,24^ The class reduction was performed per a CoD line to minimize the number of targets so the model could sufficiently learn. The targets were sorted to identify the top four CoD cases: human immunodeficiency virus (HIV) (520 entries), respiratory tuberculosis (TB) (210 entries), malaria (161 entries) and non-transport accidents (157 entries), distributed at 6.1%, 24.5%, 8.1% and 5.0%, respectively. Data augmentation was performed using the Synthetic Minority Over-sampling Technique for Nominal features (SMOTE-N) and reached a class size of 1296 each.

### Symptom cause information (SCI)

SCI is used to cater to the need for VA CoD determination. SCI is the compiled relationship between symptoms and CoD that is used by ML algorithms as a base to build CoD determination models.^5^ This study used this approach, where the SCI was computed from physicians’ assignments as the gold standard. To minimise bias, each death code by a physician was compared with a second physician; when results differed, a third physician was used to decide the final CoD. The standard CoD codes from the WHO were used. The death codes are herein referred to as class labels.

### Data preprocessing

The Iringa VA dataset had numerical and categorical features with varying scales and formats. Thus standardization was performed to harmonize the features and improve the quality of the model’s performance. A standard minimum–maximum scaler was used to ensure that all features contributed proportionally during model training, where the maximum value was 1 and the minimum value was 0. One-hot encoding was performed to convert all input features to numbers, as the Bayesian network ML model does not accept formats other than numbers. Class reduction was also performed on group targets based on the similarity of features. Further, various imputations were performed to handle missing values. Complete case analysis was applied to datetime and categorical features with missingness >50%, especially where data were missing completely at random (MCAR). For features with <5% missingness and MCAR patterns, single unconditional mean imputation (SUMI) was used, with the mean for numerical features and mode for categorical ones. For variables with system-driven missingness, multiple imputation or the use of dummy variables was ruled out due to the risk of bias and poor model interpretability. Instead, splitting symptoms per underlying CoD was performed guided by symptom patterns and physician review. Splitting meant developing multiple models for computing respective CoDs. It is a costly option in terms of time and resources but is more accurate since it minimizes the size of imputed variables and resulting uncertainties.

### Significant feature selection

After data preprocessing, feature selection was conducted in two phases to retain only the most significant and non-redundant features for CoD prediction. First, a feature combination step, guided by physician expertise and Pandas profiling outputs, merged clinically related variables (e.g. positive/negative malaria test results, duration-based symptoms) to reduce redundancy, improve interpretability and reduce missingness through systematic imputation. Second, a forward selection procedure was applied, iteratively adding features that improved cross-validated accuracy and the F1 score. This process reduced the feature space to significant features, covering deceased demographics, injury/accident history, medical history, general symptoms, maternal health indicators, TB/HIV history and natural language processing (NLP)-derived narrative features, as presented in Table 2.

**Table 2.** Selected significant features for VA CoD prediction.

Series of VA questions	CoD
age, id10002, id10003, id10004, id10055	Deceased information for all CoDs
id10077, id10079, id10082, id10098, id10099, id10100	Injuries and accidents
id10120	Ill history to be used by all CoD except accident/injury
id10120, id10123, id10125, id10126, id10127, id10130, id10131, id10132, id10133, id10134, id10135, id10136, id10137, id10138, id10139, id10140, id10141, id10142, id10143, id10144, malaria	Medical history to be used by all CoD except accident/injury
id10147, id10149, id10150, id10151, id10152, id10153, id10154, id10155, id10156, id10157, id10159, id10161, id10165, id10166, id10167, id10170, id10171, id10173, id10173_a, id10174, id10175, id10179_1, id10181, id10182, id10186, id10187, id10188, id10189, id10190_a, id10191, id10192, id10193, id10194, id10195, id10197, id10199, id10200, id10201, id10203, id10204, id10205, id10207, id10208, id10209, id10210, id10211, id10212, id10213, id10214, id10215, id10217, id10218, id10219, id10221, id10222, id10223, id10224, id10225, id10226, id10227, id10228, id10229, id10230, id10231, id10232, id10233, id10234, id10235, id10236, id10237, id10238, id10241, id10242, id10243, id10244, id10245, id10246, id10247, id10248, id10249, id10250, id10251, id10252, id10253, id10254, id10255, id10256, id10257, id10258, id10259, id10260, id10261, id10262, id10263, id10264, id10265, id10266, id10267, id10268, id10270, breathlessness, chestpainduration, feverduration	General signs and symptoms associated with final illness
id10294, id10295, id10296, id10297, id10299, id10300, id10301, id10302, id10304, id10305, id10306, id10307, id10310, id10312, id10313, id10315, id10316, id10317, id10319, id10320, id10321, id10322, id10323, id10324, id10325, id10328, id10329, id10330, id10331, id10332, id10333, id10334, id10337, id10338, id10339, id10340, id10342, id10343, id10344,	Maternal
id10411, id10412, id10413, id10414, id10415, id10416, id10418, id10419, id10420, id10421, id10422, id10423, id10424, id10425, id10426, id10427, id10446	TB/HIV
id10436, id1044	Features for NLP

### CoD classification

**Figure 1.**
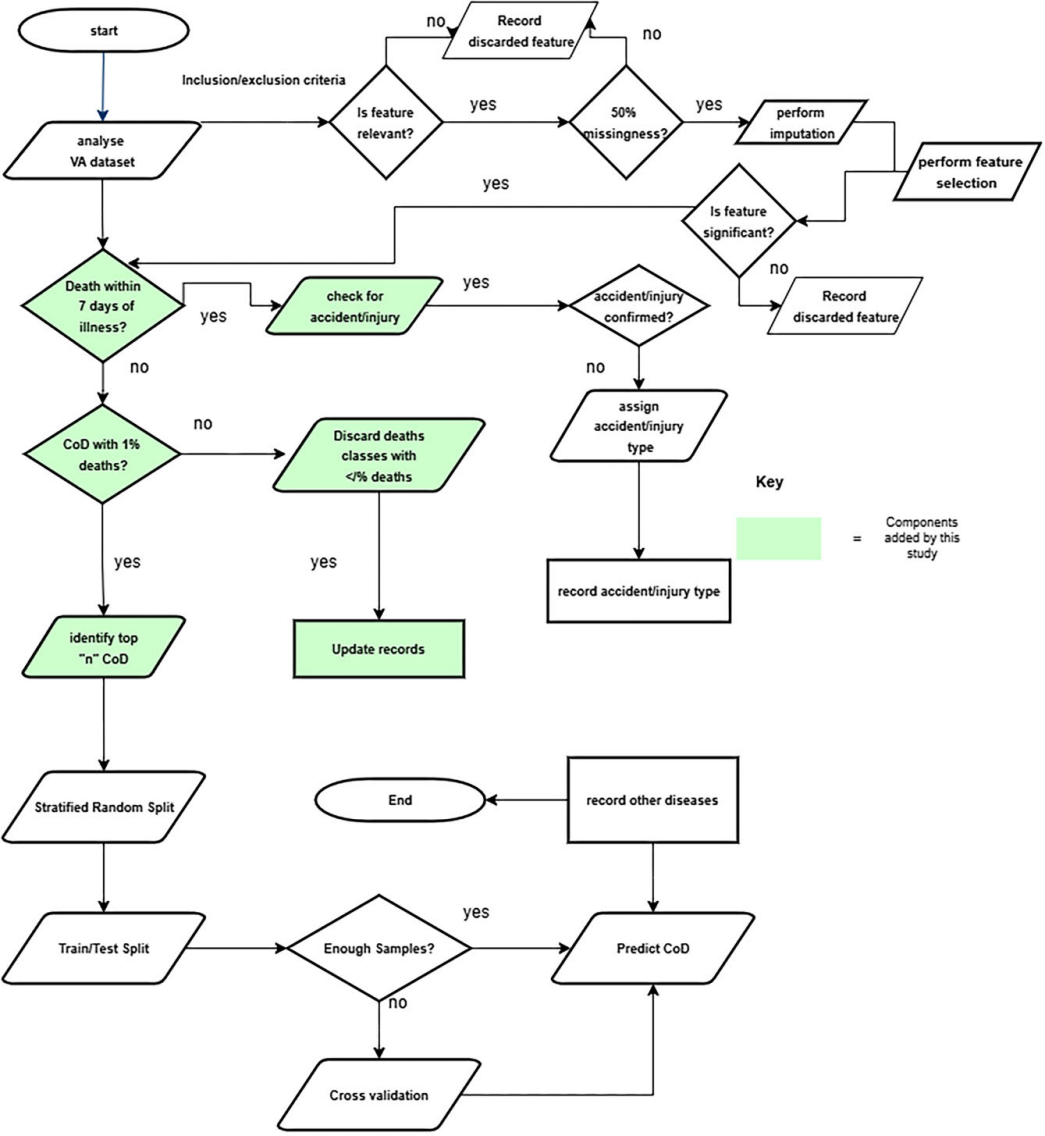
A comprehensive guide to the decision flow for predicting CoD using VA data.

**Figure 2.**
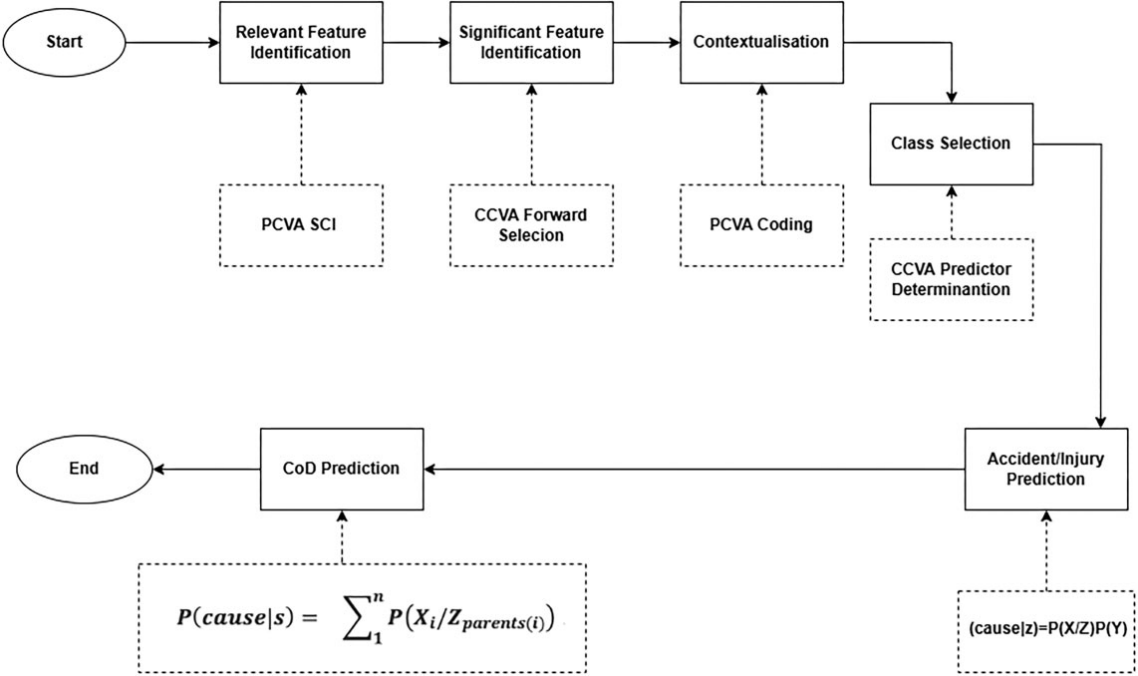
Overview of the algorithm for predicting CoD using VA data.

As presented in Figures 1 and 2, the algorithm begins by identifying relevant symptoms using SCI, and the significant features are identified through forward selection. Imputation is performed on features with <50% missingness; otherwise, features are discarded. Contextualization is done through physician expertise. After feature establishment, CoD predictors are computed. The prediction stage begins by computing the accident/injury CoD. Suppose the deceased had encountered an accident and died within the first 7 days. In that case, the conclusion is accident/injury-related death, but if it is >7 days, then it is assessed for the presence of other diseases before concluding accident/injury-related CoD. This is because the medical records suggest that 90% of patients with fatal injuries die within 7 days of admission.^25,26^ After that, the VA dataset is assessed for classes with 1% deaths and records of all classes with <1% deaths are deleted. Based on the heuristic ML approach, for multiclass output efficiency, it is recommended that each class should have at least 1% of examples of the class’s total sample size.^23,24^ The remaining cases are assessed to identify the prevailing CoD in the area by identifying the top ‘n’ CoD. To generalize the model, users will provide n as input.

The CoD classification process followed the standard Bayesian network model development, where the data are split into training/testing sets. For datasets with n>500, such as in this study (n=2509), an 80/20 train/test split is recommended and was applied. To ensure balanced class representation, 10 iterations of fivefold cross-validation were performed. Further, for multiclass outputs, it is recommended that the feature size should be around √N for highly correlated features and N−1 for less correlated features, where N is the sample size.^24^ After assessing the size of the VA dataset, CoD prediction was approached as a multiclass classification task.

The computation of the likelihood of certain diseases is conditioned on a set of symptoms. The modelling of CoD typically involves learning the relationship between a set of symptoms X that fit the presence of the disease condition E. The key aspect of Bayesian learning is assigning the prior distribution of P(E) over the symptoms X. The likelihood of the disease is computed by the conditional probability of a different subset of symptoms conditioned on E. Since for a given CoD there are subsets of symptoms, the joint probability distribution of P over the symptoms X1…Xn can be provided as a summation of all possible subsets of symptoms, as presented in equation 1.


(1) \begin{equation*}{\mathrm{P}}( {\mathrm{E}}) = \ \mathop \sum \limits_{x1 - xk} ...\, ...\mathop \sum \limits_{xk} P( {{\mathrm{X}}1......{\mathrm{Xn}}}),\end{equation*}

where X is a set of observed symptoms or signs, E is the presence of a specific disease condition (the CoD), P(E) is the prior probability distribution of the disease condition E and P(X1, X2, …, Xn) is the joint probability distribution over the symptoms or signs X1, X2, …, Xn.

In this study, a subset of the selected three CoDs is the focus; thus equation 1 is reduced by factoring out only the desired symptoms or signs related to the three CoDs. Therefore, the CoD is given by equation 2:


(2) \begin{equation*}P( {\textit{Cause}|S} ) = \mathop \sum \limits_{i = 1}^n P( {{X}_i\ |\ {Z}_{\\smallriptsize\textit{parents}( i )}} ),\end{equation*}

where Cause is the CoD, S are observed symptoms/signs leading to death, n is the total number of observations or causes being considered and Z_parents_ is the parent variables in the Bayesian model.

The three CoDs (malaria, HIV and TB) are marginally independent of each other. However, the subset of symptoms conditionally depends on the specific CoD. Some symptoms are shared among CoDs. For instance, fever is a common symptom of various diseases. However, the duration of the fever could differentiate one CoD from the other. Therefore, multiple symptoms/signs were ruled out to focus on those relevant to the specific CoD in monoclass and multiclass scenarios.

### Model implementation

The model was developed and evaluated using a standard computing environment and the Google Colab open-source cloud platform and implemented in Python; bnlearn library was used to build the Bayesian network model that was evaluated using scikit-learn. For real-world deployment, the model can be integrated into the existing data collection pipeline supported by the ODK Collect application, which is already in use by the Tanzanian MoH. Once VA data are submitted to the central servers, the trained model can be applied to generate automated CoD predictions.

### Model evaluation

The developed adaptive Bayesian network model was compared to support vector machine and naïve Bayesian models. The SVM model employed a radial basis function kernel with a regularization parameter C=1.0 and γ=0.01. For naïve Bayesian, the multinomial variant was applied with Laplace smoothing (α=1.0). Four key performance metrics were used for evaluation: accuracy, sensitivity, specificity and F1 score. These metrics are commonly applied in VA CoD classification to evaluate how well a model identifies true outcomes compared with known or physician-coded causes.^1,2,5,6^ Using these widely adopted metrics in VA CoD classification enables consistent performance benchmarking across studies.

## Results

To compare the learning ability of the Bayesian network with other ML algorithms that have been used previously to compute CoD from VA, such as SVM and naïve Bayesian, stratified cross-validation was opted for to ensure each fold had the same proportion of observations. The value of K that satisfied the evenly distributed folds was 5. Ten iterations of fivefold were performed and the results indicated average accuracy of 97%, specificity of 97%, sensitivity of 94% and F1 score of 94%. These results of the developed adaptive Bayesian networks model are superior as compared with other ML VA models tested on the same data, such as the naïve Bayesian and SVM models, which reported accuracy of 71–90% and sensitivity of 34–77%. Thus the Bayesian network algorithm was validated as a superior model for VA CoD prediction.

Two experiments were conducted to demonstrate further the capability of the Bayesian network model in predicting CoD. The first experiment was done using the original VA dataset after excluding children and maternal deaths. The second experiment was done using synthetic data after performing data augmentation in an attempt to increase and balance the dataset. For the first approach, the accuracy recorded for the model was 97.2%, with an average of 9 correctly predicted cases out of 10 cases in the Iringa VA dataset. The average sensitivity was 94.2%, the ratio of the true positives to the total positive cases. The average specificity was 97.9% and the average F1 score of 94.9%, indicating the balance between how many positive cases were correctly identified and how reliable those positive predictions were.

For the second experiment, the three CoDs (HIV, malaria and TB) selected were augmented with SMOTE-N to balance the size with the ‘other’ CoD class, which had 1296 cases, where HIV had 414 cases, TB had 168 cases and malaria had 129 cases. The augmentation was performed only on training data. To avoid biases in the test set, it was unaltered in both experiments. After performing data augmentation, all classes had the exact size of 1296. Thus the dataset was balanced.

**Figure 3.**
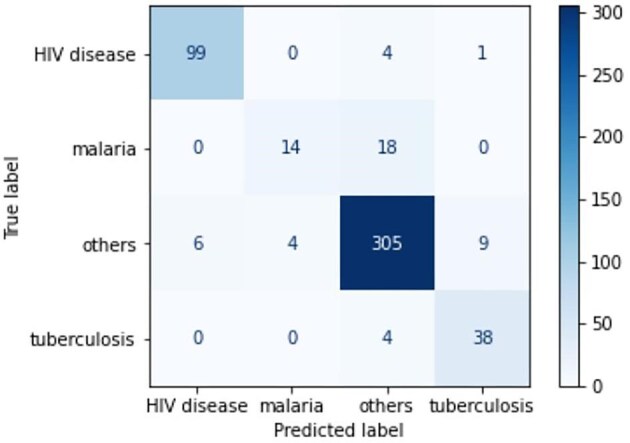
Confusion matrix for the first experiment using the original VA dataset after excluding children and maternal deaths.

**Figure 4.**
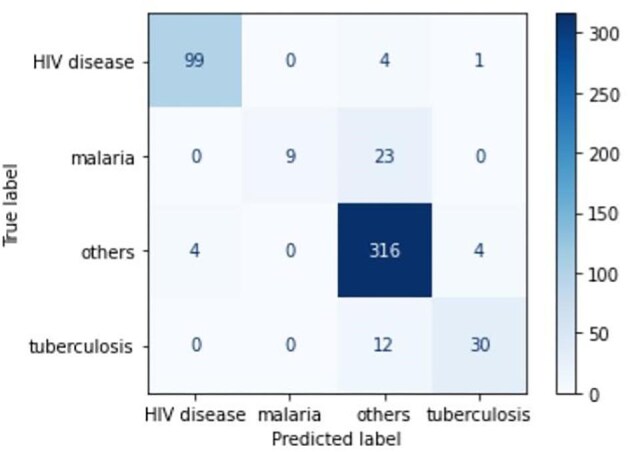
Confusion matrix for the second experiment using synthetic data after performing data augmentation in an attempt to increase and balance the dataset.

The resultant model had an accuracy of 97.8%, specificity of 98.5%, sensitivity of 95.1% and F1 score of 95.7%. The increased performance recorded is accuracy +0.6% and sensitivity +1% and +0.5%. Confusion matrices in Figures 3 and 4 present class size and sample distribution for the first and second experiments, respectively, used to compare the number of true labels between the two experiments to better understand the difference in performance.

The ‘0’ in the confusion matrix means no mislabelling error, also termed zero confusion. The diagonal presents correctly labelled values. Off-diagonal values represent misclassification on the respective class label. If the diagonal for each class label is compared, it can be observed that both experiments had similar results for HIV performance. The model produced by synthetic data performs better for the malaria and TB classes. The difference is that eight and seven more cases were labelled correctly by the model for TB and malaria, respectively. For class ‘other’, the first experiment with the original dataset performed better, with nine classes labelled correctly. Both models performed poorly on malaria classification, with a sensitivity of 44% for the second experiment and 28% for the first experiment. The main reason is that malaria symptoms are subclasses of other disease symptoms and thus pose a challenge in their computation. The difference in performance between the two experiments is accuracy +0.6% and sensitivity +1% and +0.5%, where the model with synthetic data performed slightly better in all three metrics.

## Discussion

Despite numerous studies employing ML and statistical algorithms to compute the CoD, most exhibited limited performance.^2,8–11,27^ This study sought to improve the performance of CoD prediction by developing a CoD prediction model that leverages the strengths of both ML algorithms and physician knowledge, providing a detailed CoD decision flow and integrating essential factors and steps for accurate CoD computation.

The dataset utilized in this study was obtained via the WHO questionnaire for conducting VA, similar to datasets used in previous studies. However, it is not publicly accessible but can be made available upon a reasonable request submitted to the Tanzania MoH. It is important to note that direct performance comparisons with earlier studies might be limited due to the fact that the proposed model focuses on computing four specific CoDs. This narrow scope (focusing only on four priority CoDs: accident/injury, malaria, HIV, TB) of the proposed model inherently limits its generalizability to other settings with diverse mortality patterns.

Even though SMOTE enhanced the proposed model’s performance, its application in VA data has notable limitations. First, the generation of synthetic samples using SMOTE may inadvertently amplify noise or outliers in the VA dataset.^28,29^ For instance, symptoms such as ‘prolonged fever’ or ‘jaundice’ in malaria cases are context dependent and may overlap with other causes (e.g. HIV/TB co-infection). Hence synthetic instances created without clinical validation risk distorting symptom–cause relationships, leading to overfitting and reduced generalizability. Second, SMOTE’s dependence on feature-space interpolation amplifies noise in unstructured VA narratives, particularly when minority classes are small (<50 cases).^28,29^ This aligns with findings from stroke prediction studies, where SMOTE-generated samples contradicted established clinical parameters.

The study acknowledges the risk of overfitting given the high accuracy reported after SMOTE augmentation. To mitigate this, a model complexity control was applied to restrict the number of parents per node in the Bayesian network, thus limiting the flexibility of the model and consequently decreasing the likelihood of overfitting.

Systems such as InterVA and the Population Health Metrics Research Consortium Instrument predict several CoDs but face challenges in ensuring good performance and consistency. For instance, InterVA demonstrated a κ of 0.27 in the Nairobi Demographic Surveillance Systems, and physician reviews often show sensitivity of <50% for non-communicable diseases.^30,31^ By prioritizing conditions that dominate mortality in Iringa and adhering to the recommendation of having 1% examples of the classes,^23,24^ and dropping all classes with <1%, this study’s proposed hybrid Bayes–physician model aimed to optimise precision for actionable public health decisions.

The developed Bayesian network model was trained and tested on four CoD labels, which are accident/injury, malaria, HIV and TB, prevailing cases in the Iringa region. The model performed CoD assignment with a general accuracy of 97%, specificity of 97%, sensitivity of 94% and F1 score of 94%. This is a superior performance of the model compared with naïve Bayesian and SVM, and the reported accuracy from previous studies ranged from 71% to 90% and sensitivity ranged from 34% to 77% in assigning adult individual CoDs.^10,14^

The weakness of the developed model lies on its low sensitivity for malaria. This reflects basic issues in VA-based diagnosis, particularly symptoms that overlap with other febrile illnesses and the absence of confirmatory testing. Despite the fact that this performance aligns with existing VA tools like InterVA-4, there are several opportunities for improvement. These include incorporating symptom duration thresholds (e.g. fever >72 h) and modelling dependencies between jaundice and anaemia.^32^

A possible explanation for why the Bayesian networks model performed well is its unique ability to combine expert knowledge with data to deduce complex phenomena.^16–19^ Second, its discrete nature transparently analyses multiclass phenomena, such as disease diagnosis.^19–21^ Further experiments will be conducted to incorporate the narrative part of the VA questionnaire into the developed model, aiming to improve performance and add more causes of death.

## Conclusions

This study complements the WHO’s efforts to produce quality mortality statistics. The adaptive model was developed using Bayesian networks, incorporating physician SCI as the gold standard and VA data from the Iringa region of Tanzania. This model can compute four CoDs: malaria, HIV, TB and accidents/injuries. This was mainly because of the limited availability of datasets to train the model. The developed algorithm is designed to seamlessly accommodate additional CoDs as the dataset grows, as it includes a self-check mechanism that verifies the class size before making CoD predictions. Additionally, it provides a detailed description of the VA CoD computation decision flow, serving as a guide for future researchers.

The developed hybrid Bayesian network model performed CoD assignment with an overall accuracy of 97%, specificity of 97%, sensitivity of 94% and F1 score of 94%. This performance surpasses that of the naïve Bayesian and SVM models and the reported accuracy and sensitivity from previous studies, which ranged from 71% to 90% and 34% to 77%, respectively, in assigning the CoD in adult individuals. This underscores the efficacy of combining Bayesian networks with physician SCI as a valuable tool in advancing the performance of CoD predictions.

### Limitations and future work

This study attempted to develop a model that utilizes narration and statistical features. However, the Bayesian network model performed poorly, thus narrative features were dropped. Nevertheless, the narratives had a significant influence on physician judgement, but could not provide the ML model with the ability to learn, due to inconsistency. This has been partly attributed to the limited size and poor quality of the narrations. We also faced challenges in accessing a list of Swahili stop words. Future work will explore how to include additional CoDs while retaining the interpretability and performance gains demonstrated here, as well as integrating community-level malaria transmission data to calibrate prior probabilities and explore ensemble methods to reduce misclassification of borderline cases.

In addition, this study used data from the Iringa region only, thus it lacks external validation, limiting immediate external applicability. The modelling framework can be adapted for other areas through domain adaptation techniques. Future work can include cross-region validation within Tanzania, evaluation on held-out years and, where available, incorporation of external datasets to assess generalizability across varying symptom expressions and death reporting patterns.

## Data Availability

The dataset is the property of the Tanzania Ministry of Health and is available upon reasonable request to the ministry after acquiring ethical clearance from the NIMR.
